# Supplementary material for: Invasive Pneumococcal Disease in People Living with HIV: A Retrospective Case—Control Study in Brazil
Source: Trop Med Infect Dis. 2023 Jun 19;8(6):328. doi: 10.3390/tropicalmed8060328 (PMC10304155; doi:10.3390/tropicalmed8060328)
Supplement: Supplementary file 1 [file tropicalmed-08-00328-s001.zip › tropicalmed-2405688-supplementary.pdf]

Tropical Medicine and Infectious Disease

MDPI

Supplementary Materials

Table S1. Adjusted demographic and clinical characteristics of PLWHA with IPD who died in hospital.

	Levels	Total	HR (IC95%)	*P*-value	aHR (CI95%)	*P*-value
**Al**		8				
**ICU Admission**	ICU Admission	8	Ref.	Ref.	Ref.	Ref.
**Sex at Birth**	Male	6	Ref.	Ref.	Ref.	Ref.
	Female	2	0.497 (0.1–2.46)	0.392	NC	NC
**Smoking**	Yes	5	2.143 (0.509–9.017)	0.298	NC	NC
**Drinking**	Yes	6	6.994 (1.387–35.279)	0.018	NC	NC
**Drug Use**	Cocaíne/crack	3	1.039 (0.248–4.349)	0.958	NC	NC
	Cannabis	0	NC	NC	NC	NC
**COPD**	Yes	2	0.858(0.172–4.28)	0.851	NC	NC
**DM**	Yes	0	NC	NC	NC	NC
**Liver Cirrhosis**	Yes	5	8.278 (1.972–34.756)	0.003	NC	NC
**Steroid Use**	Yes	1	3.047 (0.366–25.365)	0.302	NC	NC
**New Case HIV**	Yes	1	1.08 (0.123–9.452)	0.944	NC	NC
**ART Use**	Not	1	1.07 (0.131–8.729)	0.949	NC	NC
**CD4 (cells/mm³)**	<100	3	Ref.	Ref.	Ref.	Ref.
	100–200	2	2.425 (0.38–15.489)	0.439	NC	NC
	>200	2	0.27 (0.045–1.62)	0.152	NC	NC
**Prophylaxis for OI**	Yes	5	1.383 (0.323–5.909)	0.662	NC	NC
**Pneumo Vaccine**	Yes	0	NC	NC	NC	NC
**Respiratory Failure**	Yes	7	6.409 (0.788-52.118)	0.082	NC	NC
**IVM**	Yes	8	4977135370.714 (0–Inf)	0.998	NC	NC
**Vasoactive Drugs**	Yes	8	4977135370.714 (0–Inf)	0.998	NC	NC
**Hemodialysis**	Yes	3	4.304 (1.021–18.153)	0.046	NC	NC
**Adrenal Insufficiency**	Yes	2	9.768 (1.865–51.174)	0.006	NC	NC

PLWHA = people living with the human immunodeficiency virus /acquired immunodeficiency syndrome; IPD = Invasive pneumococcal disease; HIV = human immunodeficiency virus; COPD = chronic obstructive pulmonary disease; DM = diabetes mellitus; ART = antiretroviral treatment; OI = opportunistic infection; IVM = invasive mechanical ventilation; NC = Not Calculated. HR (CI95%) = Hazard-ratio of progression to death after hospitalization from an infection (IPD) among levels of the features related to the reference and its 95% confidence intervals estimated by Cox proportional-hazards regression models. aHR (CI95%) = HRs adjusted for confounding variables (i.e., patients' age, birth sex, race/colour, level of education, household income, Nadir T-CD4+ count, T-CD4+ count, and viral load (the latter two being measured in the last six months of follow-up) and its 95% confidence intervals estimated by Cox proportional-hazards multiple regression mode.

Table S2. Data on hospitalized PLWHA with and without IPD.

Variables	Total (*n* = 132)	IPD (*n* = 38)	Not IPD (*n* = 94)	*P*-value
**Admission to ICU**	41 (31.1%)	15 (39.5%)	26 (27.7%)	0.263
**Length of ICU Stay (days)**	8 [3.75;14]	5 [3.5;11.5]	8 [5;14]	0.327
**Respiratory Failure**	54 (40.9%)	21 (55.3%)	33 (35.1%)	0.053
**NIVM**	32 (24.2%)	10 (26.3%)	22 (23.4%)	0.897
**Invasive Mechanical Ventilation**	31 (23.5%)	11 (28.9%)	20 (21.3%)	0.833
**Acute Renal Failure**	68 (51.5%)	32 (84.2%)	36 (38.3%)	< 0.001
**Hemodialysis**	17 (12.9%)	5 (13.2%)	12 (12.8%)	1
**Vasopressor Use**	33 (25%)	11 (28.9%)	22 (23.4%)	0.657
**Cardiac Arrhythmia**	23 (17.4%)	6 (15.8%)	17 (18.1%)	0.951
**Gastrointestinal Bleeding**	11 (8.3%)	3 (7.9%)	8 (8.5%)	1
**Intracranial Mass Effect**	11 (8.3%)	0 (0%)	11 (11.7%)	0.064
**Adrenal Insufficiency**	15 (11.4%)	2 (5.3%)	13 (13.8%)	0.271

**In-Hospital Death**	22 (16.7%)	8 (21.1%)	14 (14.9%)	0.547
**Death at Last Follow Up**	63 (41.2%)	19 (42.2%)	44 (40.7%)	1

PLWHA = people living with the human immunodeficiency virus/acquired immunodeficiency syndrome; IPD = Invasive pneumococcal disease; ICU = intensive care unit; NIVM = non-invasive mechanical ventilation; *P*-values were calculated for absolute (relative) frequencies by chi-squared tests.

Table S3. Demographic and clinical characteristics of PLWHA with IPD who died in hospital.

	Levels	Total	IPD Hospital Death		*P*-value
		**38**	**Yes (*n* = 8)**	**No (*n* = 30)**	
**Age (years)**	Age	42 [35;47]	43.5 [38;51]	40 [35;46]	0.327
**Sex at Birth**	Male	28 (62.2%)	6 (75%)	22 (59.5%)	0.675
	Female	17 (37.8%)	2 (25%)	15 (40.5%)	
**Smoking**	Yes	22 (48.9%)	5 (62.5%)	17 (45.9%)	0.646
**Drinking**	Yes	16 (35.6%)	6 (75%)	10 (27%)	0.031
**Drug Use**	Cocaine/Crack	15 (33.3%)	3 (37.5%)	12 (32.4%)	0.701
	Cannabis	3 (6.7%)	0 (0%)	3 (8.1%)	
	No	27 (60%)	5 (62.5%)	22 (59.5%)	
**COPD**	Yes	13(28.9%)	2 (25%)	11 (29.7%)	1
**Liver Cirrhosis**	Yes	9 (20%)	5 (62.5%)	4 (10.8%)	0.005
**Steroid Use**	Yes	3 (6.7)	1 (12.5%)	2 (5.4%)	1
**New Case of HIV**	No	41 (91.1%)	7 (87.5%)	34 (91.9%)	1
	Yes	4 (8.9%)	1 (12.5%)	3 (8.1%)	
**CD4 (cells/mm³)**	<100	12 (27.3%)	3 (42.9%)	9 (24.3%)	0.113
	100-200	5 (11.4%)	2 (28.6%)	3 (8.1%)	
	>200	27 (61.4%)	2 (28.6%)	25 (67.6%)	
**Nadir (cells/mm³)**	Nadir	117.5 [49.25;316.75]	**53.5 [12;93.5]**	**151.5 [80.75;334]**	**0.033**
**CD4/CD8**	CD4/CD8	0.4[0.14;0.68]	0.41 [0.19;0.7]	0.39 [0.12;0.64]	0.741
**Viral Load (copies/mL)**	Detectable	793 [39;24821]	810 [39;7371.5]	751.5 [39;43780.25]	0.657
**Use of ART**	Yes	40 (88.9%)	7 (87.5%)	33 (89.2%)	1
**Prophylaxis for OI**	Yes	27 (60%)	5 (62.5%)	22 (59.5%)	1
**PPV23**	Yes	9 (21.8%)	0 (0%)	9 (24.3%)	0.284
**PCV13**	Yes	1 (2.2%)	0(0%)	1 (2.7%)	1
**ICU Admission**		15 (39.5%)	**8 (100%)**	**7 (23.3%)**	**< 0.001**
**Days in the ICU**		5 [3.5;11.5]	4.5 [2.75;6]	13 [6.5;14]	0.062
**Respiratory Failure**		21 (55.3%)	7 (87.5%)	14 (46.7%)	0.096
**NIVM**		10 (26.3%)	0 (0%)	10 (33.3%)	0.147
**IVM**		11 (28.9%)	8 (100%)	3 (10%)	< 0.001
**Acute Renal Failure**		32 (84.2%)	8 (100%)	24 (80%)	0.405
**Hemodialysis**		5 (13.2%)	3 (34.5%)	2 (6.7%)	0.088
**Vasoactive Drugs**		11 (28.9%)	**8 (100%)**	**3 (10%)**	**<0.001**
**Cardiac Arrhythmia**		6 (15.8%)	**5 (62.5%)**	**1 (3.3%)**	**<0.001**
**Adrenal Insufficiency**		2 (5.3%)	**2 (25%)**	**0 (0%)**	**0.055**

PLWHA = people living with the human immunodeficiency virus/acquired immunodeficiency syndrome ; IPD = Invasive pneumococcal disease; HIV = human immunodeficiency virus; COPD = chronic obstructive pulmonary disease; ART= antiretroviral treatment; OI = opportunistic infection; NIVM = non-invasive mechanical ventilation; IVM= invasive mechanical ventilation; PPV23 = 23 valent pneumococcal vaccine; PCV13 = 13 valent pneumococcal vaccine; AST = Aspartate aminotransferase; ALT = Alanine aminotransferase; CRP = C-reactive protein; P-values were calculated for absolute (relative) frequencies by chi-squared tests.

Table S4. Syndromes presented by PLWHA with and without IPD.

Variables	All Patients (n = 153)	IPD (n = 45)	Not IPD (n = 108)	P-value
**Respiratory Syndrome**	71 (46.4%)	36 (80%)	35 (32.4%)	< 0.001
**Neurological Syndrome**	23 (15%)	1 (2.2%)	22 (20.4%)	
**Digestive Tract Syndrome**	15 (9.8%)	6 (13.3%)	9 (8.3%)	

PLWHA = people living with the human immunodeficiency virus/acquired immunodeficiency syndrome IPD = Invasive pneumococcal disease. P-values were calculated for absolute (relative) frequencies by chi-squared tests.

Table S5. Main opportunistic diseases in PLWHA with and without IPD.

Variables	IPD		OR (CI95%)	P-value	aOR (CI95%)	P-value
	Yes (n = 45)	No (n = 108)				
**Tuberculosis**	4 (10.53%)	29 (30.85%)	0.26 (0.09–0.81)	0.020	0.45 (0.1–1.95)	0.284
**Pneumocystis Pneumonia**	3 (7.89%)	13 (13.83%)	0.53 (0.14–1.99)	0.350	1.09 (0.18–6.72)	0.926
**Neurotoxoplasmosis**	0 (0%)	10 (10.64%)	NC	NC	NC	NC
**Cryptococcal Meningitis**	2 (5.26%)	6 (6.38%)	0.81 (0.16–4.23)	0.807	1.82 (0.24–13.67)	0.562

PLWHA = people living with the human immunodeficiency virus /acquired immunodeficiency syndrome. IPD = Invasive pneumococcal disease; NC = Not Calculated. OR (CI95%) = Odds-ratio of having an infection (IPD) among levels of the features related to the reference and its 95% confidence intervals estimated by Logistic (Binomial) regression models. aOR (CI95%) = ORs adjusted for confounding variables (i.e., patients' age, birth sex, race/colour, level of education, household income, Nadir T-CD4+ count, T-CD4+ count, and viral load (the latter two being measured in the last six months of follow-up) and its 95% confidence intervals estimated by multiple Logistic (Binomial) regression models.

Disclaimer/Publisher's Note: The statements, opinions and data contained in all publications are solely those of the individual author(s) and contributor(s) and not of MDPI and/or the editor(s). MDPI and/or the editor(s) disclaim responsibility for any injury to people or property resulting from any ideas, methods, instructions or products referred to in the content.
